# Insights from one thousand cloned dogs

**DOI:** 10.1038/s41598-022-15097-7

**Published:** 2022-07-01

**Authors:** P. Olof Olsson, Yeon Woo Jeong, Yeonik Jeong, Mina Kang, Gang Bae Park, Eunji Choi, Sun Kim, Mohammed Shamim Hossein, Young-Bum Son, Woo Suk Hwang

**Affiliations:** 1 UAE Biotech Research Center, Lane 2128 Al Wathba, Al Wathba South, Abu Dhabi, UAE; 2 North Eastern Federal University, Republic of Sakha, Yakutia, Russia; 3 Department of Companion Animal and Animal Resources Science, Joongbu University, Geumsan-gun, 32713 Republic of Korea

**Keywords:** Cloning, Genetic engineering, Reproductive biology

## Abstract

Animal cloning has been popularized for more than two decades, since the birth of Dolly the Sheep 25 years ago in 1996. There has been an apparent waning of interest in cloning, evident by a reduced number of reports. Over 1500 dogs, representing approximately 20% of the American Kennel Club’s recognized breeds, have now been cloned, making the dog (*Canis familiaris*) one of the most successfully cloned mammals. Dogs have a unique relationship with humans, dating to prehistory, and a high degree of genome homology to humans. A number of phenotypic variations, rarely recorded in natural reproduction have been observed in in these more than 1000 clones. These observations differ between donors and their clones, and between clones from the same donor, indicating a non-genetic effect. These differences cannot be fully explained by current understandings but point to epigenetic and cellular reprograming effects of somatic cell nuclear transfer. Notably, some phenotypic variations have been reversed through further cloning. Here we summarize these observations and elaborate on the cloning procedure.

## Introduction

Approximately 22 animal species have been reported to be cloned by Somatic Cell Nuclear Transfer (SCNT). Among them approximately 19 have had individuals which survived to adulthood. Dolly the Sheep, cloned in 1996, is highly regarded to be the first cloned mammal. Since then, similar protocols, without substantial differences, have been followed for all other reported cloned animals.

**Figure 1:**
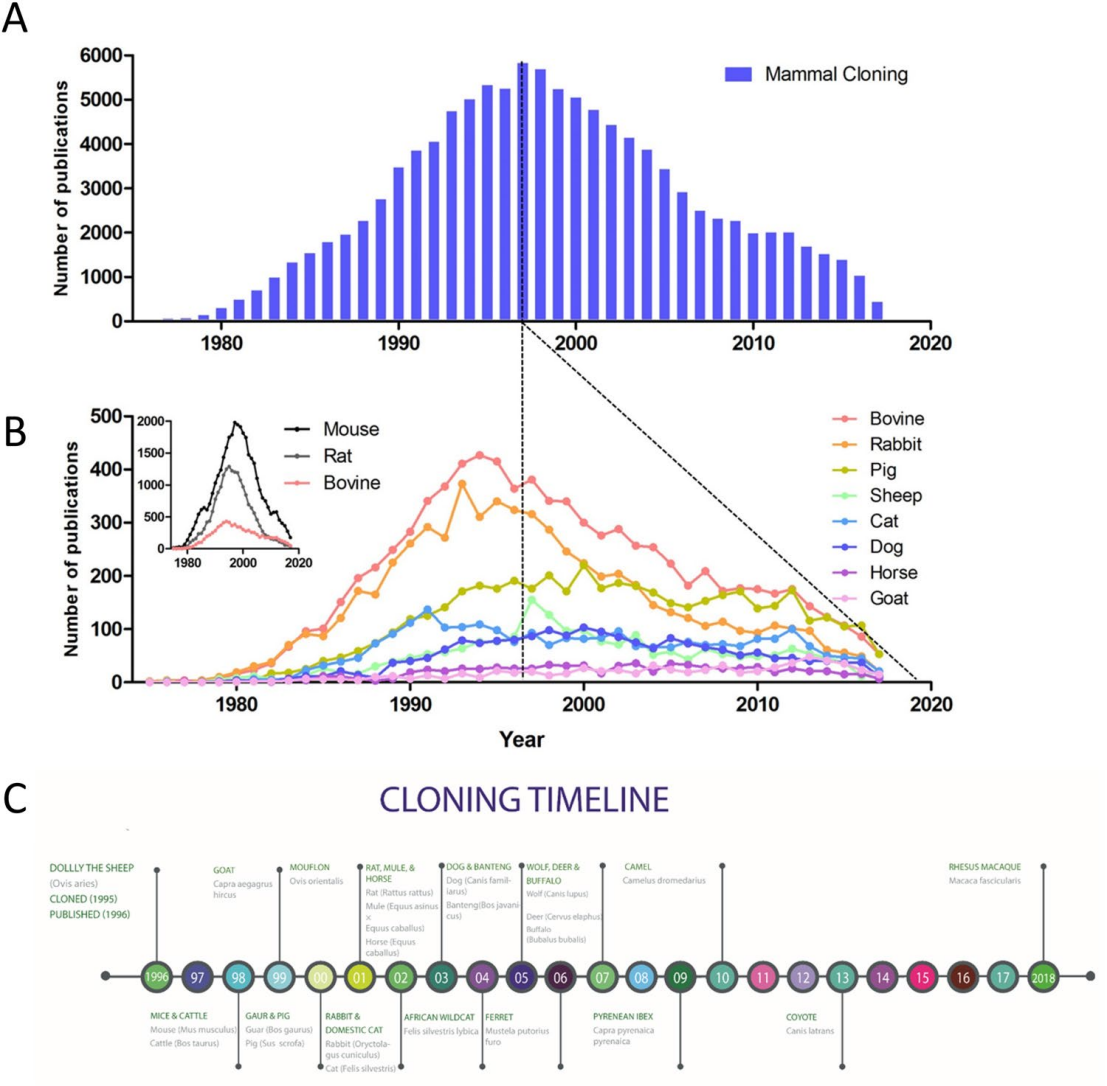
Cloning publications over time and breed differences. (**A**) Mammal cloning as represented by the number of articles published per year. (**B**) Representation of the number of publications by species per year, upper left indicates the scale difference of small laboratory animals, mice and rats, when compared to the most published of the larger species. Dotted lines indicate the peak in cloning publication as well as the declining slope of the trend from that point. (**C**) Timeline of published cloned animals, by somatic cell nuclear transfer, it should be noted that the Macaque, although included, was cloned using fetal cells.

A clear difference in the interest of animal cloning has been observed, with publications for mammal cloning reaching nearly 6000 in 1997, falling to fewer than 500 in 2017 according to PubMed (Fig. 1A,B). Why the apparent interest, based on publication number, has declined is a matter of speculation; it is not initially due to a decrease in new species cloned as the majority of species cloned were cloned in the following few years (Fig. 1C). No new species have, however, been cloned in the past 5 years. This may either be a result of cloning becoming more normalized, and thus less novelty in publication, or may represent the lack in advancement and interest it generates. As such this report aims to provide insight on canine cloning over the past two decades.

The first canine was cloned in 2005 and was the 15th animal to be cloned (Fig. 1C)^1^. Unlike other species, canine cloning remains comparatively difficult, due to the lack of in vitro oocyte maturation methods and other reproductive complexities^2^. Of the larger animals only a few, such as pigs and cattle have been reported to be cloned at a scale in the hundreds^3^.

The observations, reported herein, are based on more than 1000 cloned dogs, produced at the center over the last decade. This success is attributed to a streamlining and combination of factors, including the optimization of established techniques. To date, a total of approximately 20% of recognized dog breeds have been cloned, by us, and are reported in this study.

In spite of the difficulties related to the unique canine reproductive physiology, we have observed that factors thought to negatively influence cloning success may not be as vital as initially presumed. These factors include, among others, the breed of the cell donor, oocyte donor and surrogate, donor age and cell passage number. In fact, our data indicate a higher canine cloning efficiency than reported in most other commonly cloned species^4^.

The larger number and diversity of breeds seen in dogs surpasses that of any other animal species^5^. This has been considered being a barrier for canine reproduction between breeds. Our observations indicate that these barriers may be fewer and narrower in scope than previously assumed^6^. Intra- and Inter-species cloning efficiency differences may be further indicated or contraindicated by the ability to produce viable offspring, as we have reported^7–9^. This may enable us to answer the question to which interspecies or inter-genus cloning attempts may be successful, with reasonable application.

Additionally, although interest in publicationon cloning has declined the knowledge gained through canine cloning may benefit human medical advances due to the fact that dogs exhibit a number of shared genetic traits and diseases. More than 600 genetic defects have been reported in dogs and the number of shared genetic diseases, with man, are reported at around 350^10^. Currently for drug advancement to human clinical trials, two rodent and one non rodent animal, which includes dogs, are required^11^. Canine medical models, such as those for Alzheimer disease, diabetes, organ transplantation, drug development and psychological disorders, among others, will likely continue to be increasingly relevant into the future^12^. This further validates the importance of cloning and disease modeling for research.

## Results and discussion

### Technical improvements in canine cloning

To date we have cloned over 1500 dogs, the first 1000 puppies born are represented in this report. It is unreasonable to claim that the dog is easier to clone than other animals. On the contrary, there are at least four obvious obstacles to dog cloning. First, the number of breeds, indicating the high degree of heterogeneity, make it more difficult to select compatibility among nuclear donor, oocyte donor and surrogates. Secondly, there is insufficient knowledge about the complexities of the canine reproductive system. Thirdly, there is a lack of sufficient protocols for the successful in vitro culture of oocytes and reconstructed oocytes. Fourthly, although several research groups have attempted canine cloning, there are no known reports of further increases in methods or relationships between cloning practices and postnatal care and training.

Accordingly, the following technical improvements have been made: firstly, we selected a Korean mixed breed to obtain reliable in vivo matured oocytes of known quality. As the Korean mongrel dogs have an optimal size, they have been successfully used as surrogates for multiple breeds of various sizes. Animals are used only once for a cloning event and well cared for, with the consistent aim of decreasing animal use and reducing any potential stress or suffering.

Secondly, we matched oocyte donors with surrogates by utilizing the detection of serum hormone levels, by means of an electro-chemiluminescent immune assay^13^. The correlation between serum hormone levels and oocyte maturation status allows for determination of the appropriate time to collect in vivo matured oocytes, and the proper time to transfer reconstructed oocytes into estrous stage matched surrogates^13^. We have developed a proprietary solution for both the flushing and collection of oocytes from the oviduct as well as for the transfer of reconstructed oocytes^14^. Surrogate choice has not been shown to positively correlate with reconstructed oocytes transfer success, but there is growing evidence to support it as a factor.

Thirdly, three steps generally preformed in the cloning of other animals have been omitted^15^. These steps include the omission of any chemicals for the induction of estrus or activation of reconstructed oocytes^16^, no in vitro culture of oocytes or of reconstructed oocytes was performed and oocyte time spent ex vivo was minimized.

**Figure 2:**
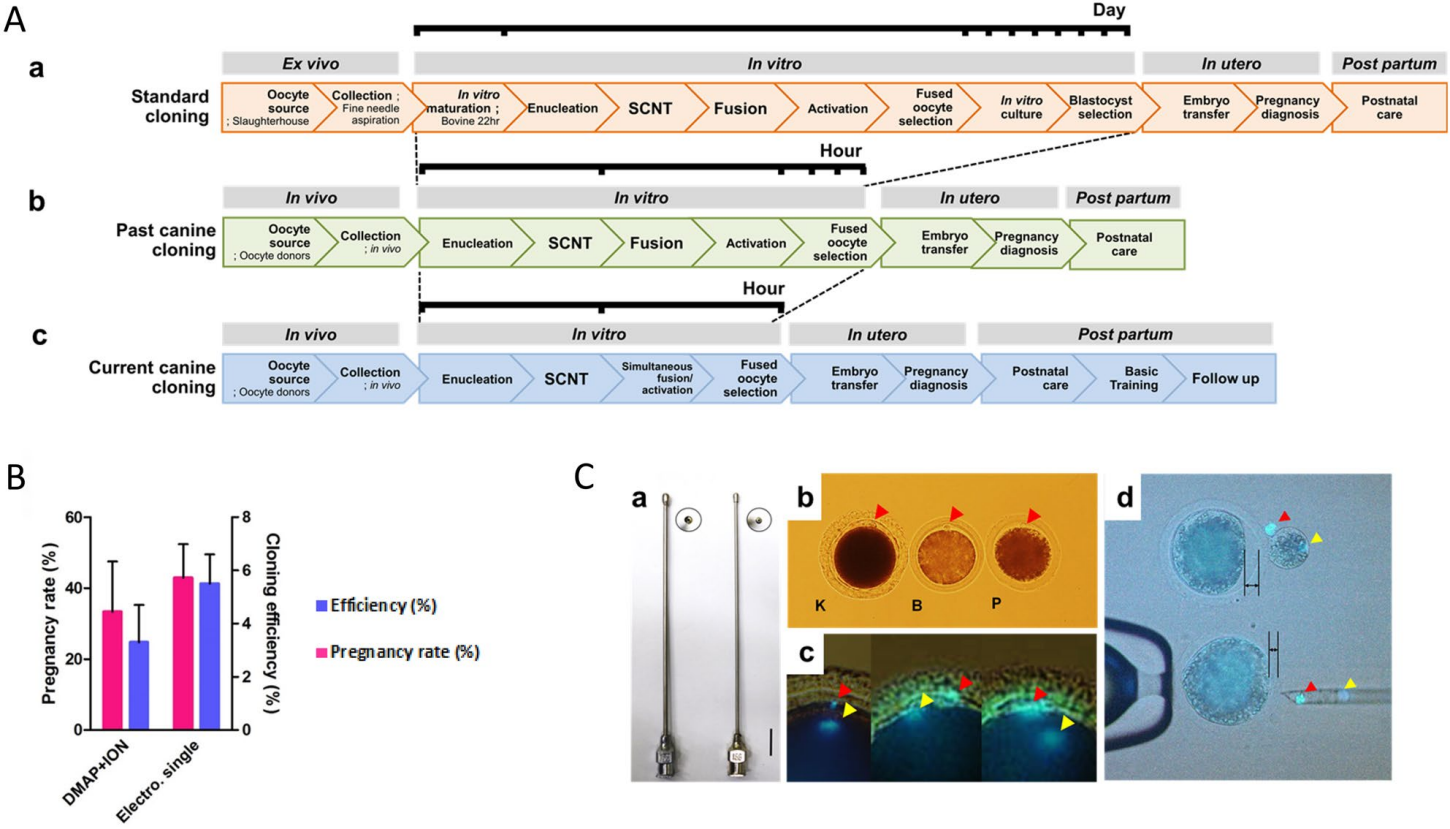
Technical improvement. (**A**) Animal reproductive cloning process; (a) a common process for bovine cloning; from the ovaries derived from the slaughterhouse, the immature oocytes are aspirated from the microneedles. These eggs become mature oocytes when cultured in in vitro maturation medium containing various hormones and growth factors for 22 h. The matured oocytes undergo a sequential process of somatic cell nuclear transfer. Afterward, the cell and cytoplasm couplets are subjected to a fusion process using electrical stimulation and then the activation of the reconstructed embryos is performed. Successfully fused cloned embryos are cultured in IVC medium for 7 days to develop into blastocysts and then transplanted into estrus synchronized surrogate mothers. (b) The method employed to produce Snuppy is still in use by other groups. Serum progesterone is measured from oocyte donors that have not undergone any hormonal treatment to determine the time of mature oocytes. The activation process is performed for 4 h after the fusion is performed. (c) Currently, we are using the method of dog cloning as an electrical stimulation to give simultaneously fusion and activation, the reconstructed embryos is directly implanted to the surrogate. (**B**) The simultaneous activation method was 1.5 times higher in cloning efficiency and 1.3 times higher in pregnancy rate although there was no significant difference due to variation. (**C**) (a) A catheter inserted into the fallopian tube, and a mature oocyte is collected by flowing a perfusion liquid through the catheter. Left 16G Right 18G. (b) Mature oocytes K = canine, B = bovine, P = porcine, the canine mature oocytes have irregular and inhomogeneous zona pelucidas compared to other species of oocytes. The cytoplasm is dark, and the nucleus and the polar body cannot be observed without Hoechst staining. (c) The nucleus of the oocyte located in the cytoplasm under the polar body is moved to the periphery of the cytoplasm when the polar body is protruded, but in canine, it is often very separated from the polar body as shown in the second and third pictures. (d) The squeezing method that we used previously is favorable for reducing damage to the cytoplasmic membrane, but cytoplasmic loss is greater than in the aspiration method.

Fourthly, several technical details have been optimized, including; the collection of oocytes by time and with a method minimizing shear stress and other factors based on volume and size of the aperture of catheters and needles used for collection and transfer. The transfer volume and number and position of post fusion transferred oocytes into the surrogate oviduct were additionally optimized. Optimized technical procedures include, among others: SCNT processes concerning enucleation, cell transfer, electric fusion and simultaneous activation of oocytes. Perhaps most importantly we designed and developed a series of methods for postnatal evaluation and care, essential to the health and wellbeing of delivered pups (Fig. 2).

The combination of the various detailed technical aspects associated with canine cloning have coalesced, by design, at this facility, enabling our success in number and repeatability of successful clones. These advancements may provide insights into cloning in general. However, we do not suggest that these improvements are universally applicable for other species.

### Cloning efficiency

Although it has been suggested that phylogenetic distance between oocyte donor, cell donor and surrogate plays a major role in cloning efficiency, calculated as the number of offspring per reconstructed oocytes^4^, ee found no significant difference in the cloning efficiency of cell donors between distantly related canine breeds^17^, although there was a large degree of variation (Fig. 2B).

**Figure 3:**
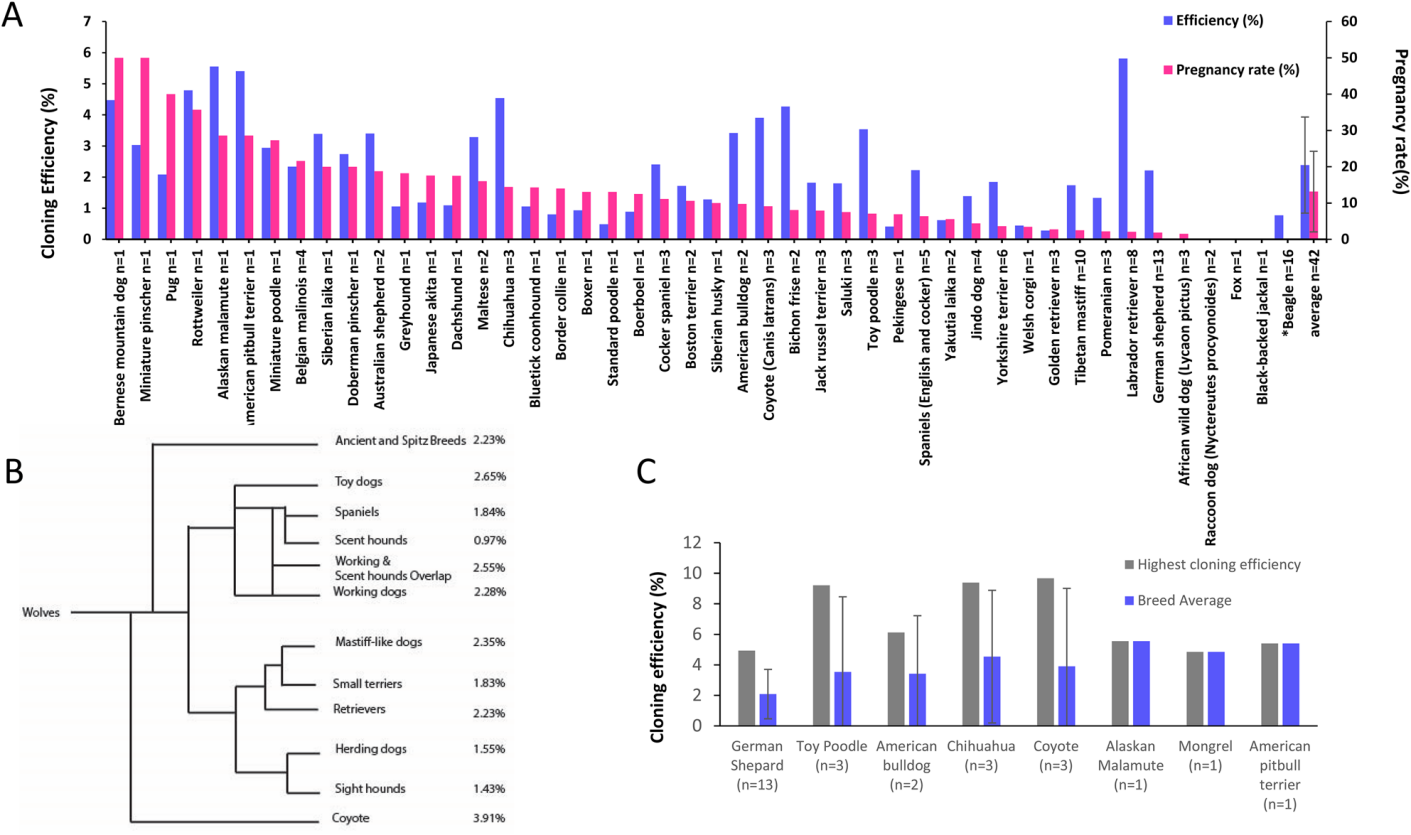
Cloning efficiency and pregnancy rate by breed. (**A**) Cloning efficiency and Pregnancy rate by individual breed. Averages displayed with STDEV. (**B**) A comparison of cloning efficiency by breed grouping, n ≥ 3 except for Scent hounds, Sight hounds and small terries which had an n = 2. (**C**) Examples of observed highest cloning efficiencies and their breed average. All error bars are standard deviation.

We observed that the greatest cloning efficiencies occurred in individuals with extreme distances between breeds, however the average cloning efficiency of these “breeds did not differ significantly from the norm”^18^. This suggests that there is a greater contribution to cloning efficiency from individual cell donors than the differences associated between breeds (Fig. 3). We report canine cloning efficiency, based on the number of live offspring produced from the number of transferred reconstructed oocytes, to be above 2.0% (Fig. 3, Supplemental Fig. 1; Sup. Table 2) which is moderately higher than that of most other reported species^19^.

It has previously been reported that seasonal variation may play a role in canine oocyte recover^20^. We however, have not observed any seasonal difference in either in oocyte number, oocyte quality or pregnancy rate. Surprisingly, oocyte-donor cell fusion rates do not appear to affect either cloning efficiency or pregnancy rates (Supplemental Fig. 1).

No differences in interbreed efficiency, with sufficient sample size, is indicated by greater genetic distance within the domestic dog and related species, such as the coyote and wolf on a modified cladogram from^17^ (Fig. 3B). More genetically distant canine species resulted in a failure of the technique. This was observed when clones of the African wild dog (*Lycaon pictus*) failed to go to term, although initial pregnancy rates were similar^9^. Other more dissimilar species, such as the red fox (*Vulpes vulpes*) and the racoon dog (*Nyctereutes procyonoides*) did not result in any detectable pregnancies (Fig. 1A). This failure is likely due to mitochondrial, immune incompatibility or other maternal–fetal barriers^9^. In spite of the apparent differences between domestic dogs and the coyote and wolf, no barriers to cloning efficiency using the domestic dog as an oocyte donor and surrogate were observed^7,8^. Due to the relative small number of cloned animal species, interspecies cloning efficiency rates are not comparable and any data, at present, must be inferred.

Efficiency is related to the number of reconstructed oocytes transferred per surrogate. Pregnancy based on the position and side and depth of transfer into the oviduct may be relevant to cloning efficiency and pregnancy success rates. We have observed that pregnancy rate but not cloning efficiency increases with the number of reconstructed oocytes transferred (Supplemental Fig. 2). One instance an unprecedented efficiency, was observed with an individual Chihuahua, (9.4%) in 32 surrogates, with an average of 16.4 oocytes transferred of which 19 surrogates delivered pups was observed (Fig. 2C). The only other individual cloned with a higher efficiency was the aforementioned coyote, in 2011, with an efficiency of 9.7%. Even including these high levels, the average cloning efficiency by breed was not significantly increased, illustrating the importance of the individual cell donor for cloning efficiency (Fig. 3, Supplemental Table 2).

Cell passage number and donor age may prove to be an important aspect in cloning efficiency, we have however insufficient data to conclusively establish whether or not there is a significant effect on either efficiency or other associated observations in cloning. The question to whether or not abnormality in offspring increases with the cell donor passage number or the age of the donor is additionally unknown, it would however appear to be contraindicated by the thousand dogs cloned in this cohort as well as work in serial cloning done in other species^21–23^.

No difference in the average cloning efficiency between breeds was observed (Fig. 3, Supplemental Table 2). A large variation in the efficiency was present, this large gap in efficiency is not well characterized and spanned breeds and species as distant as the coyote (*Canis latrans*) and the Chihuaua, examples where the difference between certain individuals, and counterparts from the same breed were most staggering (Fig. 2A,C). The coyote also exhibited a large individual to group cloning efficiency difference, with a group average of 3.91%, one individual reached 9.7%. It is postulated that certain cells are more capable of being reprogramed, although other experiments and our own observations have shown that difficult to determine^7^. This difference in cloning efficiency and phenotypic variation remains poorly characterized and without explanation, although generally assumed to be due to epigenetic reprograming failure^21,24^. Epigenetic differences in cloning warrants further investigation, which may increase cloning efficiencies, decreasing costs and potentially allowing for the cloning of various species or hard to clone individual cases.

### Postnatal survival of cloned dogs

Like humans, dogs are altricial, compared with precocial young of other species, the reasoning for this reproductive strategy is not fully understood in carnivore^25^. The postnatal developmental differences between species pose unique requirements for their care. Methods for canine postnatal care have been developed and gradually optimized, by us, over the past decade. We are now able to report only minimal losses after parturition. Significant survival rates, based on the improved care system developed in the initial years additionally adds to the increased success and stability of canine cloning. Potentially confounding, survival rates, cesarean section has been utilized to further maximize live birth rates by rescuing pups which exhibit signs of fetal distress^26–28^.

Health assessment has been performed using a modified APGAR scoring method, capable of characterizing pup survival rates with accuracy. At this point adult survival is next to guaranteed in cloned pups which are born healthy. Apart from a few cases of care related deaths (< 2%), generally attributed to the surrogate mothers, there have been no premature death or increased incidence of disease observed or reported in cloned dogs, assessed to be healthy at birth. The longevity of cloned dogs does not appear to be diminished compared to breed averages, based on observations to date. The oldest living cloned animal, as far as we know, is a cloned dog, residing in Korea, born in March of 2007.

### Phenotypic variations in cloned dogs

The cloning process allows for the observation of multiple genetically identical individuals, which may shed light on various developmental associated phenotypes. These cases may warrant further investigation into similar human conditions as they relate to the phenomena observed in cloned dogs. Occasionally phenotypic variations are observed from genetically identical individuals following the cloning process^29^. Here we describe several such observations from individuals from the 1000 + clones produced over the last 10+ years (Supplemental Table 1; Sup. Fig. 3).

*Sex reversal* has been observed in several cloning cases, originating from 10 different breeds representing one fourth of the total number of breeds cloned to date^30,31^. Although not widely investigated it has additionally been observed in natural births^32^. In sex reversed clones, from German Shepherds, the major male sex determinant gene, *SRY*, was found to be present in all the clones as well as the donors. Interestingly, it was both hyper-methylated in the somatic donor tissue as well as in individuals which exhibited the sex reversed phenotype, from that donor. The hyper-methylated status correlated to gene expression and was without detectable mutation or sequence variation. The frequency of sex reversed clones was decreased in the same donor cell population after treatment with a demethyltransferase inhibitor, 5-Aza-2′-deoxycytidine (AZA)^22^. Offspring originating from AZA treated donor cells exhibited varied growth rates, compared with their untreated cohorts. This deviation in growth is likely due to epigenetic alteration based on methylation of regulatory genes^11^. This causes us to question the genotype of naturally born pups with a female phenotype. It is not known at which frequency this occurs in nature, or if it is influenced by breed, as appears to occur in cloning^33^, as we have observed an increased incidence of sex reversal in clones from German Shepherd cell donors. The relative few number of individuals cloned per breed prevents adequate comparison in this regard. No incidence of female to male sex reversal has been observed in the more than one thousand cloning cases to date.

*Heterochromia Iridis*, also known as odd eye, is a disparity between the eye color of an individual. This has been observed in a number of clones and has not inferred any detectable negative health issues. Its cause and regulation are not well established^34^. It has been proposed that there may be association with a condition referred to as Mirrored Appearance, but this has not yet been verified in our research.

*Microphthalmia*, a condition associated with uni- or bi-lateral ocular under development, has been observed from two cell donors, with four affected individuals in this series. There are multiple factors associated with the development of the eye and its surrounding structures^35^. No causal relationship has been identified from these cases.

*Cleft palate* is an abnormal phenotype identified in many human individuals^36^. The phenotype is associated with a variety of genetic mutations showing diverse inheritance patterns in man^37^.

Our studies show that, in some genetically identical cloned animals, cleft palate may occur a rate of 2.9%. This is in accordance with human medical observations from twin studies, where cleft palate occurs at a higher frequency from non-genetic causes than inherited ones. This is evident by a greater number of discordant affected monozygotic pairs than mutually affected ones^38^. This phenotype has been observed in a few cloned animals, born amongst unaffected cloned littermates. When the phenotype has been observed, in more than half of the cases (58.33%) it has been presented alongside other malformations (41.67% cleft palate alone). The conclusion that this phenotype arises from non-genetic causes is further supported by porcine cloning data where serial cloning has rescued the phenotype^22^.

*Large offspring syndrome* (LOS) is known to occur in various methods of Assisted Reproductive Technology including in vitro fertilization (IVF) in humans and animals and animal cloning^39^. It has been suggested to be a model for study of the human Beckwith–Wiedemann syndrome (BWS)^40^. In cloned animals, LOS has been observed in, among others, pigs, sheep and cattle^41^.

*Macroglossia*, a condition observed where the tongue is disproportionately large, is correlated with decreased postnatal survival, due to the difficulty in feeding or respiration^42^. The incidence of macroglossia alone was 0.97% of live born pups in our data set. We have observed normal development from individuals diagnosed with a mild phenotype. The causal mechanism behind these variations is not fully understood^43^.

*Muscular hypermyotrophy* is a condition exhibiting abnormal overdevelopment of the muscle^44^. Hypermyotrophy alone occurred in 4.95% of all cases from our data set. However, it is interesting to note that a combination of hypermyotrophy and macroglossia occurred in 9.78% of all cases. Not all individuals which exhibited muscular hypermyotrophy were accompanied with macroglossia. The muscular hypermyotrophy observed was nearly always fatal. It appears that difficulties in respiration are the primary cause for these deaths. There has been interest in increased muscle mass mutations, which can be beneficial^45^. Further study into the mechanism behind this observed variation is warranted.

It is thought that these peculiarities in the cloning process are due to incomplete cellular reprograming, resulting from the methylation or genetic regulated state of the donor cell^46^. Cell type and origin, of the donor, may be associated with these formations^47^. We agree that the evidence shows an epigenetic mechanism associated with the reprograming process as described above, treatment with the methylation inhibitor, AZA, reduced phenotypic abnormalities when administered prior to SCNT.

## Conclusion

Canine cloning has matured over the last decade, in part due to refinement of cloning techniques. A relatively low number of phenotypic variations have been reported. No abnormalities related to longevity of healthy born clones have been identified. Future potential applications of animal cloning continues to have the potential for reproductive rescue of endangered and extinct species. Cloned animals may additionally provide information for human medical insights. The successful cloning of dogs in increasing number has indicated the increased general interest regarding cloning and the general outlook of cloning appears to be shifting towards acceptance. Several practical and ethical questions persist and should be continually evaluated with consideration for both advantages and disadvantages related to the various use of animals. Cloning in the technological toolkit of biology will undoubtedly continue to play a role in the production and conservation of canids and other species into the future.

## Materials and methods

### Care and use of animals

Standard animal care protocols were followed as previously established (Ref. P4 paper^13^. Briefly, all female mixed breed dogs were between 1 and 7 years old (body weight 20–25 kg). Animals were housed in indoor kennels, fed standard commercial dog food once a day, and given water ad libitum. All animal procedures were conducted in accordance with the animal study guidelines and approved by the committee at the Sooam Biotech Research Foundation, Korea (permit no. C-12-01) and within Animal research: reporting of in vivo experiments (ARRIVE) guidelines. Informed consent was obtained for of all animals included in this study.

### Ovulation determination

All other reagents were obtained from Sigma-Aldrich. All oocyte donors and recipients in the study were showing spontaneous estrous. The estrous stage was examined weekly by observing for vulvar bleeding which indicates the onset of the heat period. During heat, 2 mL of blood sample was collected daily by cephalic venipuncture and serum P4 levels were measured by electrochemiluminescence immunoassay (ECLIA; Cobas e411, Roche Diagnostics, Mannheim, Germany; intra- and inter-assay coefficients of variation < 4%). Ovarian ultrasonographies were performed twice a day when serum P4 levels were found to rise more than 2 ng/mL. Time of ovulation was designated when ovaries become difficult to find for an apparent decrease in the number or contour of anechoic follicles, or for their disappearance anechogenicity by transabdominal ultrasonography and as the proportion of cornified cells were greater than or equal to 90% of epithelial cells from vaginal swabs, stained following Diff Quik (Sysmex Co., Kobe, Japan) standard protocols^48^.

### Oocyte collection

All oocyte donors and surrogates underwent spontaneous estrous, donors and surrogates were matched based on their synchronization of estrus. Oocytes were surgically retrieved 3–4 days post ovulation. Before surgery, a blood sample was drawn through the cephalic venipuncture, and the blood plasma was collected and frozen (− 20 °C) for hormone analyses. Anesthesia was induced with a mixture of xylazine hydrochloride (Rumpun^®^; Bayer Korea, Ansan, Korea; 1 mg/kg body weight) and ketamine HCl (Ketalar^®^; Yuhan Corporation; 50 mg/mL, Seoul, Korea; 4 mg/kg body weight) and maintained with isoflurane inhalational. Under aseptic conditions, the reproductive tract was exposed through a midventral incision. Oocytes were bilaterally flushed from each oviduct with 10 mL TCM 199 supplemented with HEPES (Invitrogen Corporation, Carlsbad, CA). Oocytes were collected using a stereomicroscope and transferred to fresh medium until undergoing SCNT.

### Evaluation of retrieved oocytes

The maturation stage of the retrieved oocytes was determined as previously described^49^. The oocytes were stripped of cumulus cells and pre-stained with 5 mg/mL bisbenzimide (Hoechst 33342) to visualize the presence of nuclei for enucleation process. The oocytes were graded based on morphology and nuclear stage as immature (cumulus very closely attached to oocytes, nuclear stage is either germinal vesicle (GV), GV breakdown, or metaphase I), mature (M II oocytes with several layers of cumulus cells and homogeneous cytoplasm), aged (unidentified nuclear status with the cytoplasmic membrane shrink, MII oocytes with less than 70% of cytoplasm and loosely attached cumulus cells,), abnormal (irregular cytoplasmic contour, protrusion of zona pellucida, nuclear immaturity), or ruptured (oocytes with broken zona and cytoplasmic membrane) under an inverted microscope equipped with epifluorescence (TE2000-E; Nikon Corporation, Japan).

### Preparation of donor cells

Donor cells originated form dogs active in police or military service. Dermal tissue samplesmeasuring approximately 1 × 3 cm were collected under light tranquilization (Zoletil 50^®^ Virbac, SA, Carros, France) at 0.1 mg/kg and local anesthesia (Daehan lidocaine HCl 2%, Dai Han Pharm Co Ltd, Seoul, Republic of Korea), or post mortem. Sections of the subcutaneous tissues were cut into small pieces (approximately 1 × mm^2^) and were cultured in Dulbecco modified Eagle medium (DMEM, Life Technologies, Rockville, MD) + 10% fetal bovine serum (FBS, Life Technologies, Rockville, MD) at 37 °C in an atmosphere of 5% CO2 and air to obtain fibroblasts. Explants were maintained in the culture until they approached 90% confluence. Cells were then trypsinised and reconstituted at concentrations of approximately 1 × 106 cells per mL, then cryopreserved in cryovials containing DMEM + 20% FBS + 10% dimethyl sulfoxide (DMSO).

### Nuclear transfer

After the evaluation of the maturation status, metaphase II oocytes were enucleated by squeezing out the first polar body and the metaphase II plate into a small amount of surrounding cytoplasm using a glass pipette. Donor cells were prepared and treated through a conventional system of primary cell culture as described previously^50^. Using a fine pipette, a trypsinized fetal fibroblast with smooth cell surface was transferred into the perivitelline space of an enucleated oocyte. The couplets were equilibrated with 0.26 M mannitol solution containing 0.5 mM of HEPES, 0.1 mM of CaCl2 and MgSO4 for 4 min. After that, the couplets were transferred to a chamber with two electrodes and covered with mannitol solution. The couplets were fused with two DC pulses of 1.75–1.85 kV/cm for 15 μs using a BTX Electro-Cell Manipulator 2001 (BTX, Inc., San Diego, CA, USA). After simultaneous fusion and activation, a group of 5–6 embryos were cultured in 25 μL microdrops of mSOF covered with mineral oil for 1 h at 39 °C in a humidified atmosphere (5% O_2_, 5% CO_2_ and 90% N_2_) until embryo transfer.

### Embryo transfer and pregnancy diagnosis

Surrogate dogs with estrus matching that of oocyte donors were anaesthetized as described earlier in oocyte retrieval procedure. The ovary with a greater number of corpus lutuea was approached from a ventral laparotomy. The fat layer covering the ovary was gently grasped with forceps and suspended with a suture to exteriorize the fimbriated end of the oviduct. As soon as fusion and activation were completed, all reconstructed embryos were loaded into a tomcat catheter (3.5 Fr × 5.5″; Severeign, Sherwood, USA) with at least a medium volume (2–4 μl) and gently transferred into the 2/3 distal position of oviduct through infundibulum.

Pregnancy was confirmed using transabdominal ultrasound with a real-time ultrasonograph 25–30 days after embryo transfer. Ultrasonography was performed either in standing or dorsal recumbency position using a portable ultrasound machine with a 3.5 MHz curved transducer (Sonace R7; Samsung Medison, Seoul, Korea). Ultrasonographies were repeated every 7 days on pregnant surrogates until term. The sizes and shapes of the chorionic cavities and the presence of an embryonic or fetal heartbeat were examined to identify embryonic or fetal death.

## Care protocols

### Sanitation

Careful consideration is given for the care and sterilization of the instruments, area and personnel where the surrogate dogs and cloned puppies are kept. Cloned puppies and surrogate dogs are all taken care in a secluded and isolate area to minimize exposure. All caretakers clean their hands and all exposed areas with commercially available sanitizers before entering and exiting the designated areas. Exterior area is cleaned and sterilized once a day using commercially available cleaning and disinfectant solutions. All housing is cleaned once or twice a week with commercially available cleaning solutions while incubators are cleaned once a day when not in use. All dishware and utensils are washed daily to prevent foodborne illnesses.

### Environment

All housing for the surrogate dogs and cloned puppies have internal heating systems to establish comfortable living environments, including central environmental control. Overall, ambient temperature is set to 25–27 °C and humidity at 50–60%. Housing floor heating is set to 35 °C but may be changed accordingly to match the seasonal requirements. Lighting was managed to provide a continuous cycle. In the evening, low voltage dim lights were used to minimize disturbance. Basic sound-proofing as well as the prevention of loud noises was implemented to minimize or prevent any unnecessary stress to surrogates or puppies. Surrogates and pups were kept together and socialized daily in addition to 24-h supervision.

### Body weight control

For the first 14 days after the birth, the cloned puppies are fed 8 times a day and from there on they are fed 6 times a day. The amount of milk provided to the puppies varies with the increase of their body weight. (logic behind milk feeding). The puppies are weaned at the age of 6 weeks, after which they are fed dry feed, the number of times and amount varying according to their age. Careful records were kept of their weight gain to monitor their condition. Up to 6 weeks of age, all cloned puppies were weighed every 3 h. A 10% increase of body weight in within 24 h was considered to be ideal. If body weight decreased by 10%, compared to their weight of the previous day, additional methods were undertaken to remedy weight loss. Sweetened barley tea may be fed to the cloned puppy using a 1 ml syringe every 2 h. Generally, when no increase in body weight is observed a first attempt may be made to assist in feeding naturally from the surrogate mother. If feeding is not efficient enough bottle feeding of the surrogate’s milk or milk replacer may be employed. Should that fail direct feeding by a syringe and finally a feeding tube may be implemented if necessary, to prevent weight loss and stimulate weight gain.

### Body temperature control

During the first week after birth, the temperature is measured once a day. We have established a temperature scale with which to compare the condition of the puppies. During the first week their body temperature should be within 35–37.2 °C and 36.1–37.8 °C starting from their second week and up to their third. From the fourth week on, their temperature should stay between 37 and 38.5 °C. A temperature below 37 °C is considered hypothermic, 38.6–39.5 °C is considered a mild fever, 39.6–40 °C is a medium fever and higher than 40 °C is a high fever. There is continuous monitoring as well as tactile temperature verification to ensure that the puppies are not sick. Should the temperatures fall from the normal range, the caretakers take measures to treat the puppy.

#### Data reporting

No statistical methods for the predetermination of sample size, were performed, statistical analysis was only preformed on groups or between individuals consisting of three or more members, unless otherwise noted. Researches were not blinded during experimentation or data analysis.

### Donor oocytes

Oocytes were obtained from anesthetized Korean mixed breed bitches, with their estrus stage determined by external indication and the exact timing for oocyte collection by serum progesterone concentration^13^. Oocytes were transferred, at physiological temperature, to a heated platform where they were assessed and selected based on their quality and maturation status. Ooctyes were stained, using Hoechst and enucleated by the aspiration method^49^.

### SCNT

Canine cells were obtained from living or recently deceased individuals, or from cells banked by third party companies. Primary cell culture was established using either explant or collagenase treatment methods. Cells were allowed to grow to confluence or near confluence, without the addition of serum starvation or other cell synchronization techniques. Cells were harvested by trypsination and following tryspin inactivation, were held in SCNT buffer. Cells with a smooth exterior and of adequate size were inserted into enucleated oocytes^49,51^. Following activation, reconstructed oocytes were transferred into stage matched surrogates, exhibiting spontaneous estrus^49^.

### Pregnancy diagnosis

Twenty-eight to 30 days post transfer of reconstructed oocytes, surrogates were given an ultrasound to determine pregnancies^52^.

### Ethics approval and consent to participate

All animal procedures were conducted in accordance with the animal study guidelines and approved by the committee at the Sooam Biotech Research Foundation, Korea (permit no. C-12-01) and within Animal research: reporting of in vivo experiments (ARRIVE) guidelines. All data presented from animals for which there is consent to participate.

### Consent for publication

The authors consent to the publication of this manuscript, which may include, the details within the text.

## Supplementary Information

Supplementary Information 1.

Supplementary Information 2.

## Data Availability

Data and materials available upon reasonable request through the corresponding author.

## Abbreviations

APGAR: Appearance, pulse, grimace, activity, and respiration
AZA: 5-Aza-2′-deoxycytidine
DMSO: Dimethyl sulfoxide
DMEM: Dulbecco’s modified Eagles medium
FBS: Fetal Bovine Serum
GV: Germinal vesicle
IVF: In vitro fertilization
IVM: In vitro maturation
SCNT: Somatic cell nuclear transfer

## References

[1] Lee BC (2005). Dogs cloned from adult somatic cells. Nature.

[2] Lange-Consiglio A (2017). Oviductal microvesicles and their effect on in vitro maturation of canine oocytes. Reproduction.

[3] Oback B, Wells DN (2003). Cloning cattle. Cloning Stem Cells.

[4] Keefer CL (2015). Artificial cloning of domestic animals. Proc. Natl. Acad. Sci. USA.

[5] Leroy G (2011). Genetic diversity, inbreeding and breeding practices in dogs: Results from pedigree analyses. Vet. J..

[6] Summers KM (2014). Limited genetic divergence between dog breeds from geographically isolated countries. Vet. Rec..

[7] Hwang I (2013). Successful cloning of coyotes through interspecies somatic cell nuclear transfer using domestic dog oocytes. Reprod. Fertil. Dev..

[8] Kim MK (2007). Endangered wolves cloned from adult somatic cells. Cloning Stem Cells.

[9] Son YB, Jeong YI, Chan Hwang K, Jeong YW, Hwang WS (2021). Mitochondrial metabolism assessment of lycaon-dog fetuses in interspecies somatic cell nuclear transfer. Theriogenology.

[10] Sargan DR (2004). IDID: Inherited diseases in dogs: Web-based information for canine inherited disease genetics. Mamm. Genome.

[11] Administration, F. A. D. (ed U.S. Department of Health and Human Services) (2005).

[12] Gurda BL, Bradbury AM, Vite CH (2017). Canine and feline models of human genetic diseases and their contributions to advancing clinical therapies. Yale J. Biol. Med..

[13] Kim JJ (2017). Relationship between time post-ovulation and progesterone on oocyte maturation and pregnancy in canine cloning. Anim. Reprod. Sci..

[14] Kim MK (2005). Effects of estradiol-17beta and progesterone supplementation on in vitro nuclear maturation of canine oocytes. Theriogenology.

[15] Li GP, White KL, Bunch TD (2004). Review of enucleation methods and procedures used in animal cloning: State of the art. Cloning Stem Cells.

[16] Smith LC, Yoo JG (2009). Animal cloning by somatic cell nuclear transfer. Methods Mol. Biol..

[17] Parker HG, Gilbert SF (2015). From caveman companion to medical innovator: Genomic insights into the origin and evolution of domestic dogs. Adv. Genom. Genet..

[18] Jeong YW (2014). Influence of somatic cell donor breed on reproductive performance and comparison of prenatal growth in cloned canines. Theriogenology.

[19] Yanagimachi R (2002). Cloning: Experience from the mouse and other animals. Mol. Cell Endocrinol..

[20] Hossein MS (2007). Influence of season and parity on the recovery of in vivo canine oocytes by flushing fallopian tubes. Anim. Reprod. Sci..

[21] Hwang KC (2009). Depigmentation of skin and hair color in the somatic cell cloned pig. Dev. Dyn..

[22] Cho SK (2007). Serial cloning of pigs by somatic cell nuclear transfer: Restoration of phenotypic normality during serial cloning. Dev. Dyn..

[23] Wakayama S (2013). Successful serial recloning in the mouse over multiple generations. Cell Stem Cell.

[24] Long CR, Westhusin ME, Golding MC (2014). Reshaping the transcriptional frontier: Epigenetics and somatic cell nuclear transfer. Mol. Reprod. Dev..

[25] Noonan MJ, Newman C, Buesching CD, Macdonald DW (2015). Evolution and function of fossoriality in the carnivora: Implications for group-living. Front. Ecol. Evol..

[26] Moon PF, Erb HN, Ludders JW, Gleed RD, Pascoe PJ (1998). Perioperative management and mortality rates of dogs undergoing cesarean section in the United States and Canada. J. Am. Vet. Med. Assoc..

[27] Melandri M, Alonge S, Peric T, Bolis B, Veronesi MC (2019). Effects of alfaxalone or propofol on giant-breed dog neonates viability during elective caesarean sections. Animals (Basel).

[28] Ramachandrappa A, Jain L (2008). Elective cesarean section: Its impact on neonatal respiratory outcome. Clin. Perinatol..

[29] Cibelli JB, Campbell KH, Seidel GE, West MD, Lanza RP (2002). The health profile of cloned animals. Nat. Biotechnol..

[30] Jeong YH (2016). Stochastic anomaly of methylome but persistent SRY hypermethylation in disorder of sex development in canine somatic cell nuclear transfer. Sci. Rep..

[31] Hwang KC (2020). Demetylation of the sex-determining region Y gene promoter and incidence of disorder of sex development in cloned dog males. J. Physiol. Pharmacol..

[32] Bennett CP (1993). Deletion 9p and sex reversal. J. Med. Genet..

[33] Schelling C, Pieńkowska A, Arnold S, Hauser B, Switoński M (2001). A male to female sex-reversed dog with a reciprocal translocation. J. Reprod. Fertil. Suppl..

[34] Imesch PD, Wallow IHL, Albert DM (1997). The color of the human eye: A review of morphologic correlates and of some conditions that affect iridial pigmentation. Surv. Ophthalmol..

[35] Böse J (2004). The phosphatidylserine receptor has essential functions during embryogenesis but not in apoptotic cell removal. J. Biol..

[36] Dudas M, Li WY, Kim J, Yang A, Kaartinen V (2007). Palatal fusion - where do the midline cells go? A review on cleft palate, a major human birth defect. Acta Histochem..

[37] Dixon MJ, Marazita ML, Beaty TH, Murray JC (2011). Cleft lip and palate: Understanding genetic and environmental influences. Nat. Rev. Genet..

[38] Ooki S (2013). Concordance rates of birth defects after assisted reproductive technology among 17 258 Japanese twin pregnancies: A Nationwide survey, 2004–2009. J. Epidemiol..

[39] Hill JR (2014). Incidence of abnormal offspring from cloning and other assisted reproductive technologies. Annu. Rev. Anim. Biosci..

[40] Chen Z, Robbins KM, Wells KD, Rivera RM (2013). Large offspring syndrome: A bovine model for the human loss-of-imprinting overgrowth syndrome Beckwith-Wiedemann. Epigenetics.

[41] Young LE, Sinclair KD, Wilmut I (1998). Large offspring syndrome in cattle and sheep. Rev. Reprod..

[42] Cohen MM (2005). Beckwith-Wiedemann syndrome: Historical, clinicopathological, and etiopathogenetic perspectives. Pediatr. Dev. Pathol..

[43] Prada CE, Zarate YA, Hopkin RJ (2012). Genetic causes of macroglossia: Diagnostic approach. Pediatrics.

[44] Hong IH (2011). Morphological abnormalities, impaired fetal development and decrease in myostatin expression following somatic cell nuclear transfer in dogs. Mol. Reprod. Dev..

[45] Mosher DS (2007). A mutation in the myostatin gene increases muscle mass and enhances racing performance in heterozygote dogs. PLoS Genet..

[46] Wee G (2007). Epigenetic alteration of the donor cells does not recapitulate the reprogramming of DNA methylation in cloned embryos. Reproduction.

[47] Tian XC, Kubota C, Enright B, Yang X (2003). Cloning animals by somatic cell nuclear transfer–biological factors. Reprod. Biol. Endocrinol..

[48] Johnston, S. D., Kustritz, M. V., & Olson, P. S. *Canine and feline theriogenology* (Saunders, 2001)

[49] Hossein MS (2009). Birth of Beagle dogs by somatic cell nuclear transfer. Anim. Reprod. Sci..

[50] Hossein MS (2009). Cloning missy: Obtaining multiple offspring of a specific canine genotype by somatic cell nuclear transfer. Cloning Stem Cells.

[51] Hossein MS (2008). Protocol for the recovery of in vivo matured canine oocytes based on once daily measurement of serum progesterone. Cloning Stem Cells.

[52] Kim S (2009). Production of cloned dogs by decreasing the interval between fusion and activation during somatic cell nuclear transfer. Mol. Reprod. Dev..

