# Supplementary material for: Insights from one thousand cloned dogs
Source: Sci Rep. 2022 Jul 1;12:11209. doi: 10.1038/s41598-022-15097-7 (PMC9249891; doi:10.1038/s41598-022-15097-7)
Supplement: Supplementary file 1 — Supplementary Information 1. [file 41598_2022_15097_MOESM1_ESM.pptx]

## Slide 1
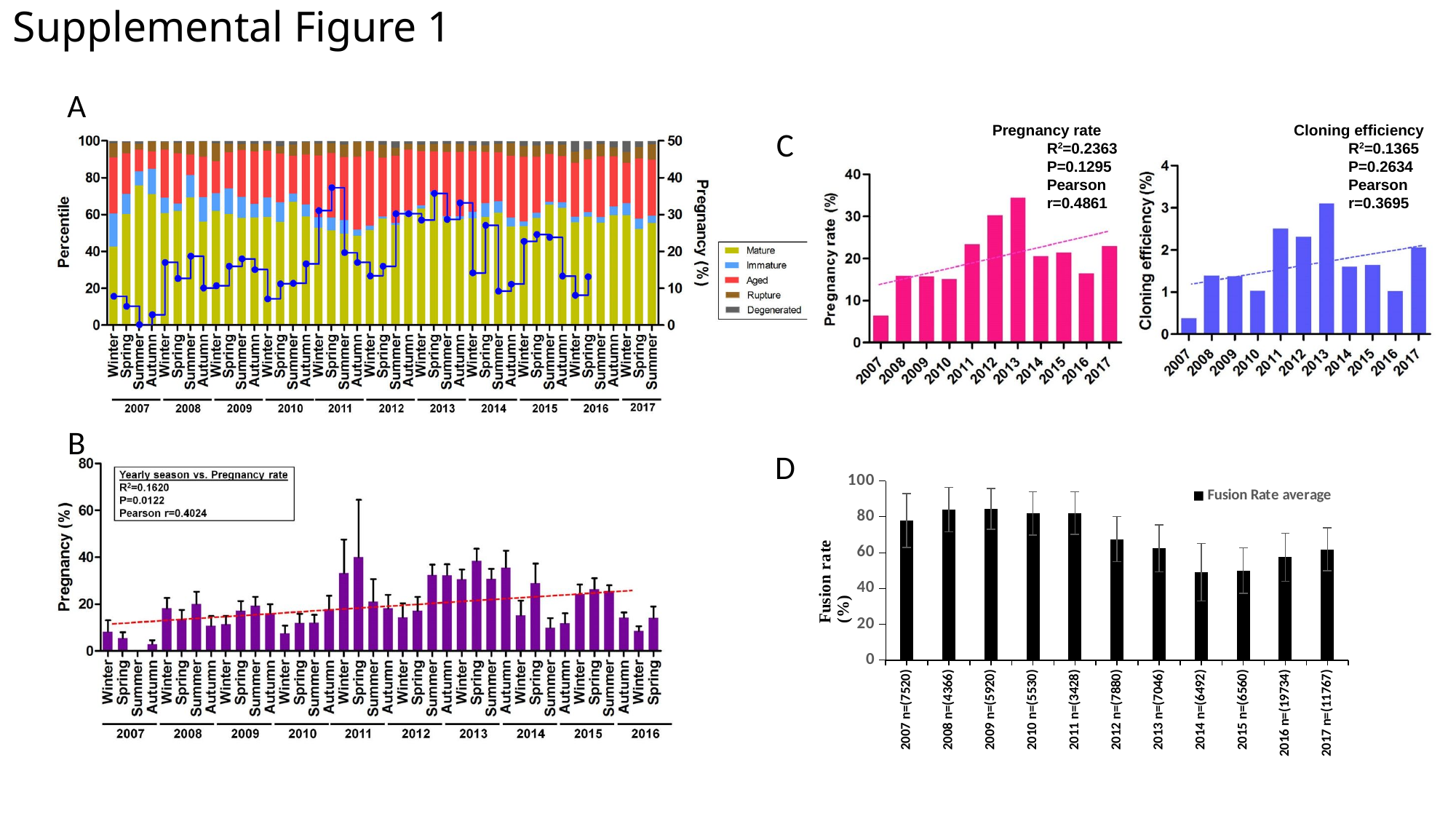

# Supplemental Figure 1
A
Pregnancy rate
R2=0.2363
P=0.1295
Pearson r=0.4861
Cloning efficiency
R2=0.1365
P=0.2634
Pearson r=0.3695
C
B
D
### Chart
| Category | Fusion Rate average |
|---|---|
| 2007 n=(7520) | 77.91647193738301 |
| 2008 n=(4366) | 84.11114215481938 |
| 2009 n=(5920) | 84.47695426251416 |
| 2010 n=(5530) | 81.90736830529114 |
| 2011 n=(3428) | 82.13442521586529 |
| 2012 n=(7880) | 67.55425835730739 |
| 2013 n=(7046) | 62.488649676022966 |
| 2014 n=(6492) | 49.07693512780443 |
| 2015 n=(6560) | 49.98084017309646 |
| 2016 n=(19734) | 57.449920907091155 |
| 2017 n=(11767) | 61.81101650307507 |

## Slide 2
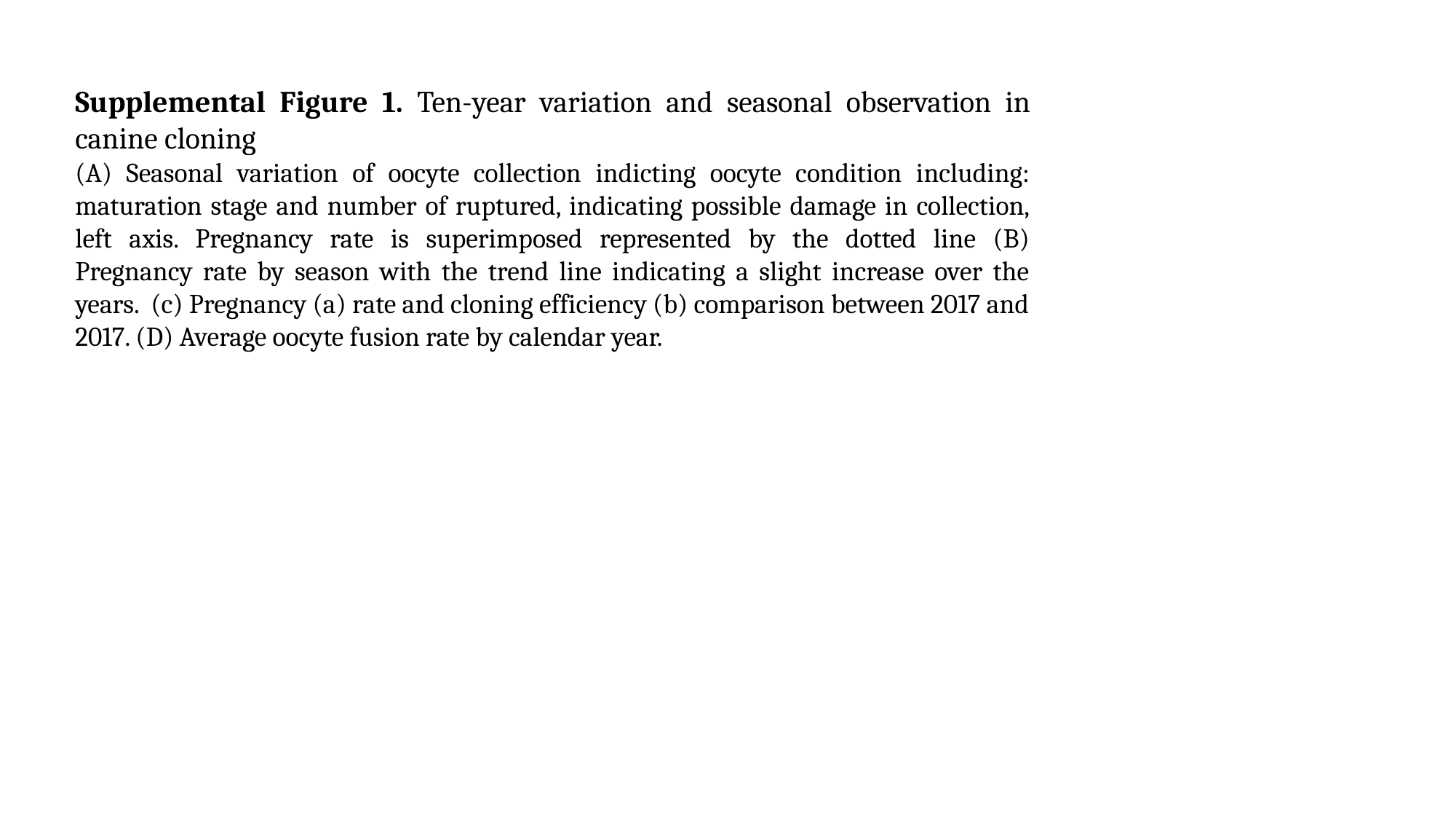

Supplemental Figure 1. Ten-year variation and seasonal observation in canine cloning
(A) Seasonal variation of oocyte collection indicting oocyte condition including: maturation stage and number of ruptured, indicating possible damage in collection, left axis. Pregnancy rate is superimposed represented by the dotted line (B) Pregnancy rate by season with the trend line indicating a slight increase over the years. (c) Pregnancy (a) rate and cloning efficiency (b) comparison between 2017 and 2017. (D) Average oocyte fusion rate by calendar year.

## Slide 3
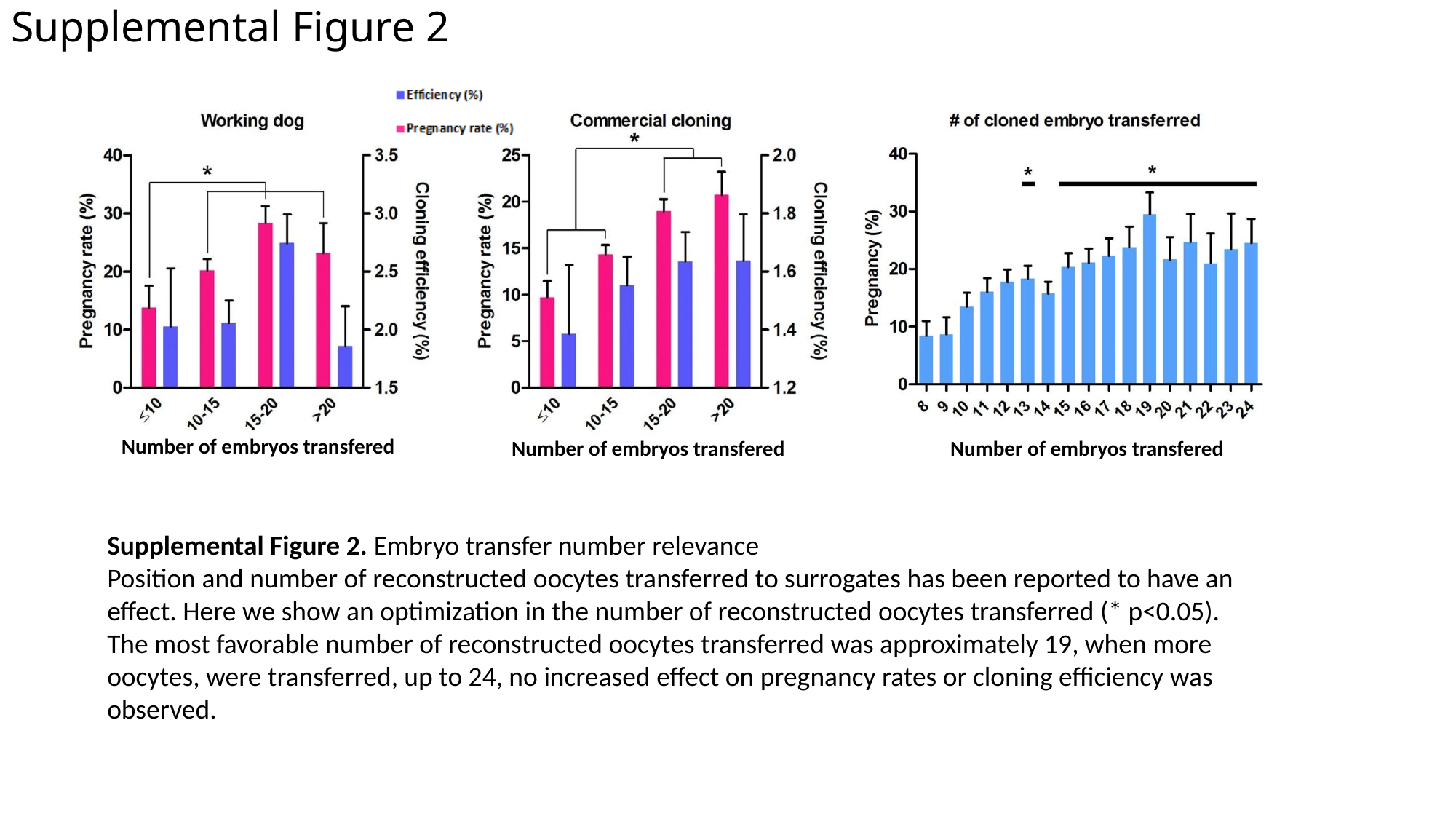

Supplemental Figure 2
Number of embryos transfered
Number of embryos transfered
Number of embryos transfered
Supplemental Figure 2. Embryo transfer number relevance
Position and number of reconstructed oocytes transferred to surrogates has been reported to have an effect. Here we show an optimization in the number of reconstructed oocytes transferred (* p<0.05). The most favorable number of reconstructed oocytes transferred was approximately 19, when more oocytes, were transferred, up to 24, no increased effect on pregnancy rates or cloning efficiency was observed.

## Slide 4
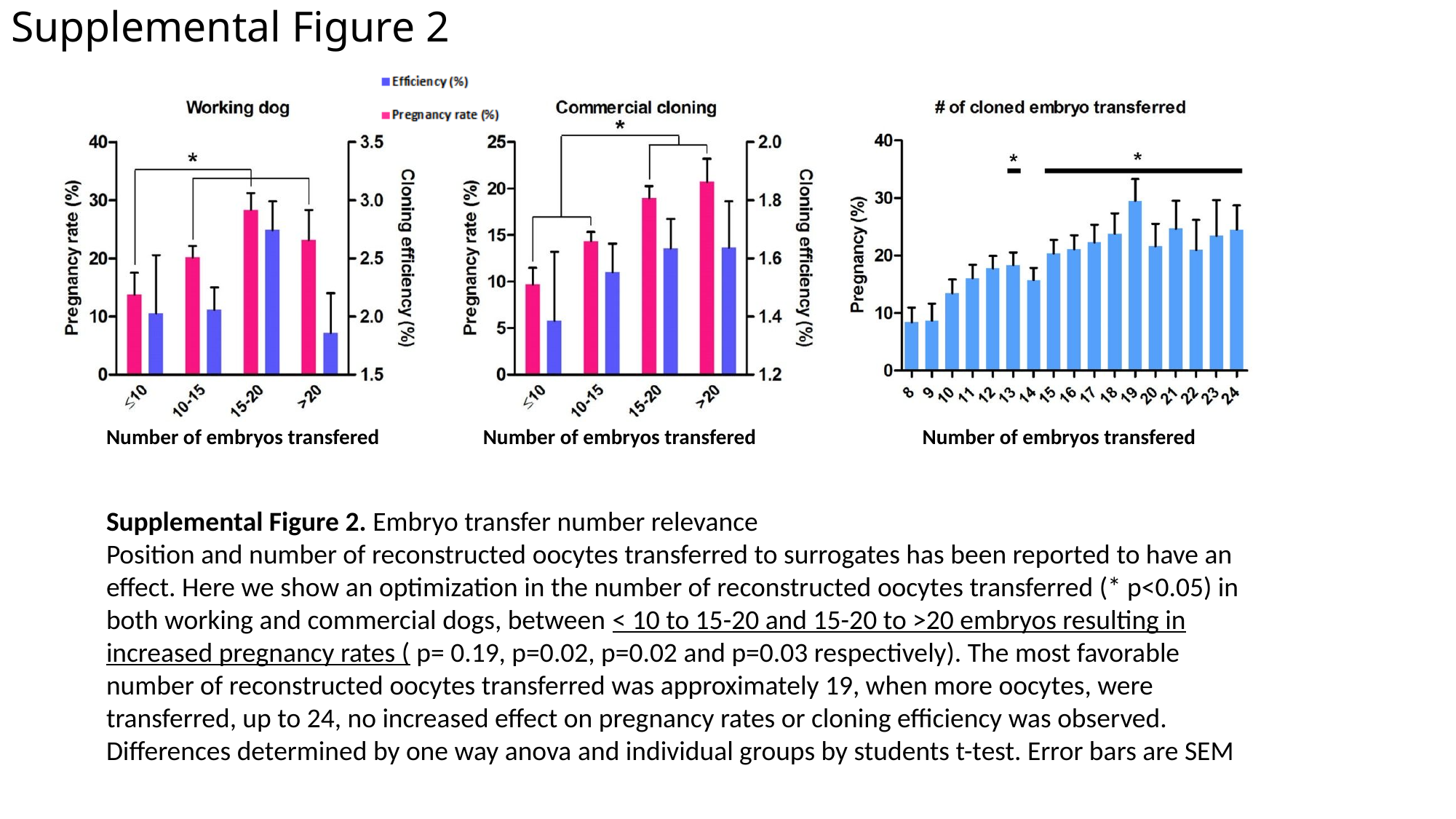

Supplemental Figure 2
Number of embryos transfered
Number of embryos transfered
Number of embryos transfered
Supplemental Figure 2. Embryo transfer number relevance
Position and number of reconstructed oocytes transferred to surrogates has been reported to have an effect. Here we show an optimization in the number of reconstructed oocytes transferred (* p<0.05) in both working and commercial dogs, between < 10 to 15-20 and 15-20 to >20 embryos resulting in increased pregnancy rates ( p= 0.19, p=0.02, p=0.02 and p=0.03 respectively). The most favorable number of reconstructed oocytes transferred was approximately 19, when more oocytes, were transferred, up to 24, no increased effect on pregnancy rates or cloning efficiency was observed. Differences determined by one way anova and individual groups by students t-test. Error bars are SEM

## Slide 5
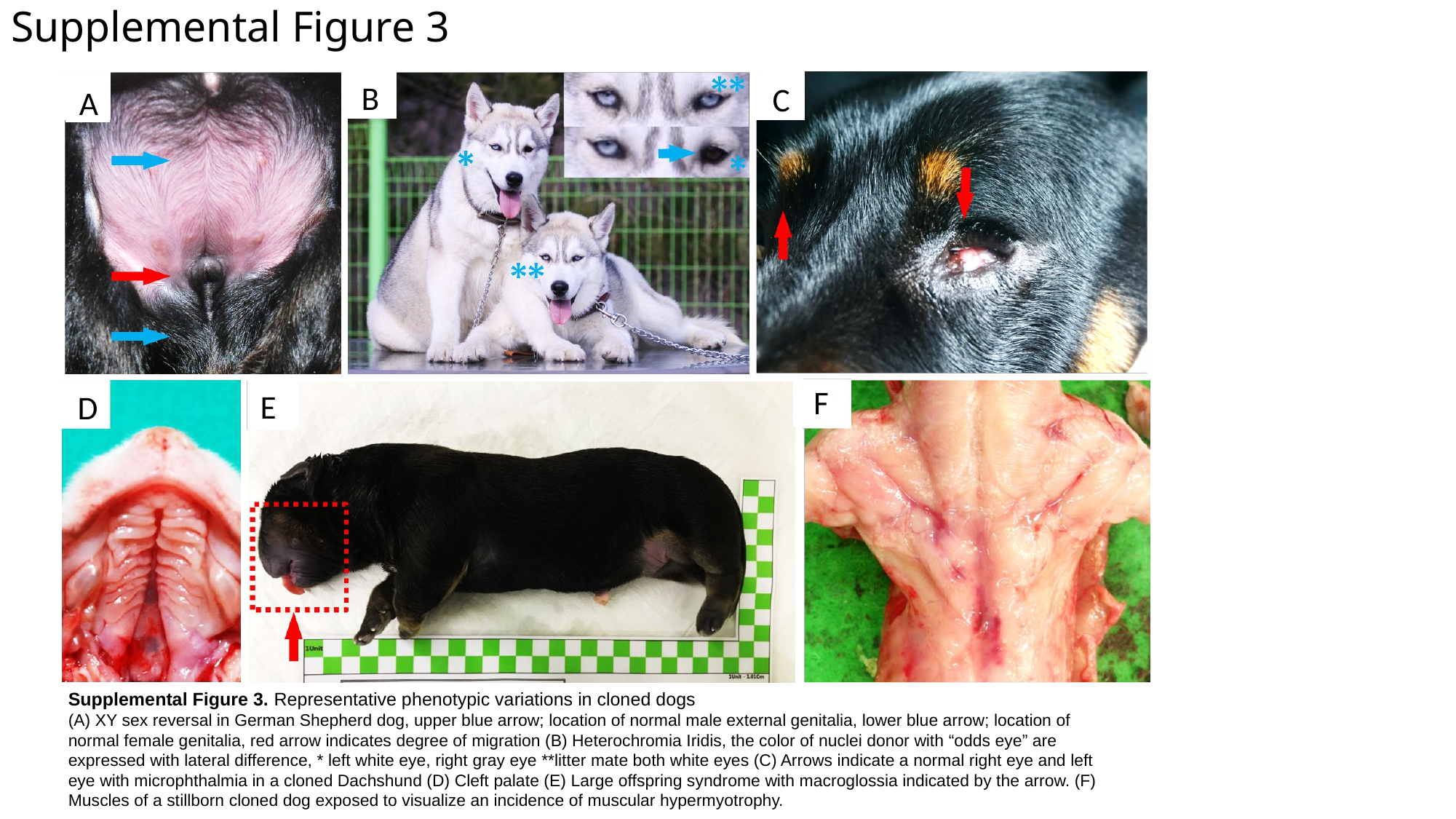

Supplemental Figure 3
B
C
A
F
D
E
Supplemental Figure 3. Representative phenotypic variations in cloned dogs
(A) XY sex reversal in German Shepherd dog, upper blue arrow; location of normal male external genitalia, lower blue arrow; location of normal female genitalia, red arrow indicates degree of migration (B) Heterochromia Iridis, the color of nuclei donor with “odds eye” are expressed with lateral difference, * left white eye, right gray eye **litter mate both white eyes (C) Arrows indicate a normal right eye and left eye with microphthalmia in a cloned Dachshund (D) Cleft palate (E) Large offspring syndrome with macroglossia indicated by the arrow. (F) Muscles of a stillborn cloned dog exposed to visualize an incidence of muscular hypermyotrophy.
