# Supplementary material for: Insights from one thousand cloned dogs
Source: Sci Rep. 2022 Jul 1;12:11209. doi: 10.1038/s41598-022-15097-7 (PMC9249891; doi:10.1038/s41598-022-15097-7)
Supplement: Supplementary file 2 — Supplementary Information 2. [file 41598_2022_15097_MOESM2_ESM.docx]

Supplemental Table 1. Phenotypic variation in clones

| Condition | Percentage of Cloned Pups (%) (Total <20%) | Usually Fatal | Total n= | Included in Research | Spontaneous Observation |  | Frequency among Variations (%) |  |
| --- | --- | --- | --- | --- | --- | --- | --- | --- |
| Macroglossia ^a,b^ | 10.75 | Yes | 109 | Yes | Yes |  | 56.19 |  |
| Hypermyotrophy ^a,b^ | 14.73 | Yes | 145 | Yes | Yes |  | 74.74 |  |
| Cleft pallet ^a^ | 2.89 | Yes | 25 | Yes | Yes |  | 12.89 |  |
| Sex Reversa ^a, c^ | 3.02 | No | 26 | Yes | Yes |  | 13.40 |  |
| Microphthalmia ^a, c^ | 0.48 | No | 4 | Yes | Yes |  | 2.06 |  |
| Hydrops fetalis ^a^ | 1.02 | Yes | 2 | No | Yes |  | 1.03 |  |
|  |  |  |  |  |  |  |  |  |
| Macroglossia alone ^c^ | 0.97 | No | 11 | Yes | Yes |  | 5.67 |  |
| Hypermyotrophy alone ^c^ | 4.95 | Yes | 47 | Yes | Yes |  | 24.23 |  |
| Macroglossia + Hypermyotrophy | 9.78 | Yes | 98 | Yes | Yes |  | 50.52 |  |
|  |  |  |  |  |  |  |  |  |

Supplemental Table 1. List of observed non-genetic variations obtained through development following SCNT. Numbers do not represent the actual spontaneous frequency observed; several variations were obtained from individuals with known increased probability as well as from cells which had been frozen without cryoprotectants. Several of the listed phenotype variations were observed only in specific cell lines, or from specific conditions, i.e. when cells were obtained from post mortem frozen tissue. Conditions are reported as total incidence and may include more than one observed phenotype per individual ^a^. The phenotype was general a compound one exhibiting more than one variation ^b^. Condition may not have any observable deleterious effect ^c^.

Supplemental Table 2. Breed and cloning efficiency by group.

| Grouping | Individual Breeds | Breed efficiency average (%) | Group average (%) | Standard deviation |
| --- | --- | --- | --- | --- |
| Ancient and Spitz | Alaskan Malamute  Siberian Laika  Jindo dog  Siberian husky  Japanese Akita  Yakutia Laika | 5.6 3.4 1.4 1.3 1.2 0.6 | 2.2% | 1.88 |
| Scent Hounds | Dachshund* Beagle* Bluetick Coonhound | 1.1 0.77 1.1 | 1.0 | 0.17 |
| Scent/Working Dogs | Toy Poodle Miniature Poodle Standard Poodle Miniature Pincher Doberman Pincher | 3.5 2.9 0.5 3.0 2.7 | 2.5 | 1.19 |
| Working Dogs | German Shepherd* Belgian Malinois | 2.2 2.3 | 2.3 | 0.09 |
| Herding dogs | Australian Shepherd Welsh Corgi Border Collie | 3.4 0.4 0.8 | 1.5 | 1.62 |
| Retrievers | Rottweiler Beninese Mountain dog Golden Retriever Labrador Retriever | 4.8 4.5 0.3 5.8 | 2.2 | 1.88 |
| Mastiff Like | Boxer Boston terrier Tibetan Mastiff American pitbull terrier Boerboel American bulldog | 0.9 1.7 1.7 5.4 0.9 3.4 | 2.3 | 1.75 |
| Toy Dogs | Chihuahua Pug Pomeranian Pekinese  Bichon fries Maltese | 4.5 2.1 1.3 0.4 4.3 3.3 | 2.7 | 1.65 |
| Spaniels | Springer Spaniel English Cocker Spaniel American Cocker Spaniel | 2.4 2.2 0.9 | 1.8 | 0.83 |
| Small Terriers | Yorkshire Jack Russel | 1.8 1.8 | 1.8 | 0.02 |
| Sight hounds | Saluki  Greyhound | 1.8 1.1 | 1.4 | 0.53 |
| Coyote | Coyote | 3.9 | - | - |
| Total average | All breed average | 2.3 | **1.97** | **1.54** |

Supplemental Table 2.
Breed Group and individual breed cloning efficiency averages and standard deviation within the group. Represented Breed efficiencies are averages of individual averaged efficiencies. * Breeds where experimental methods were included which may have affected efficiency reports.
